# Clinical and Electrodiagnostic Correlations of Ultrasound-Detected Markedly Enlarged Median Nerve at the Wrist

**DOI:** 10.3390/neurolint17080124

**Published:** 2025-08-07

**Authors:** Lisa B. E. Shields, Vasudeva G. Iyer, Theresa Kluthe, Kahir Jawad, Jun Cai, Yi Ping Zhang, Christopher B. Shields

**Affiliations:** 1 Norton Neuroscience Institute, Norton Healthcare, Louisville, KY 40202, USA; yipingzhang50@gmail.com (Y.P.Z.); cbshields1@gmail.com (C.B.S.); 2 Neurodiagnostic Center of Louisville, Louisville, KY 40245, USA; pavaiyer@gmail.com; 3 Norton Research Institute, Norton Healthcare, Louisville, KY 40202, USA; theresa.kluthe@louisville.edu (T.K.); kahir.jawad@nortonhealthcare.org (K.J.); 4 Department of Pediatrics, University of Louisville School of Medicine, Louisville, KY 40202, USA; jun.cai@louisville.edu

**Keywords:** carpal tunnel syndrome, markedly enlarged median nerve, electrodiagnostic study, ultrasonography

## Abstract

Background/Objectives: This is a retrospective review of 36 patients with electrodiagnostic (EDX) confirmation of carpal tunnel syndrome (CTS) and ultrasound (US) detection of marked median nerve enlargement (defined as a cross-sectional area [CSA] of 40 mm^2^ or greater) at the wrist. Methods: We describe the clinical, electrodiagnostic (EDX), and US findings in these patients and discuss the pathophysiologic basis of a markedly enlarged median nerve. Results: The markedly enlarged median nerve was detected by US in a total of 39 hands (36 patients, with 3 bilateral). Of the 39 hands, thenar atrophy was observed in 15 (38.5%) hands, and pinprick loss in the median nerve distribution was noted in all hands. Moderately severe or severe median nerve entrapment at the carpal tunnel (CT) was confirmed by EDX studies in 21 (53.8%) and 16 (41.0%) hands, respectively. A total of 12 (30.8%) hands had no compound muscle action potentials (CMAPs) over the abductor pollicis brevis muscle, and sensory nerve action potentials (SNAPs) were not detected in 31 (79.5%) hands. The wrist CSA was between 40 and 44 mm^2^ in 20 (51.3%) hands, between 45 and 49 mm^2^ in 13 (33.3%) hands, and 50 mm^2^ or greater in 6 (15.4%) hands. Conclusions: The implications of the markedly enlarged median nerve for surgical management of CTS are unknown, and future prospective studies are needed.

## 1. Introduction

Ultrasound (US) has become an important complementary, and in some circumstances essential, tool in confirming carpal tunnel syndrome (CTS) [1,2]. US is a simple, cost-effective, rapid, readily available, accurate, and noninvasive technique to visualize the median nerve and its surrounding anatomical structures [3]. Additionally, it may reveal a space-occupying lesion, an anatomical variation of the median nerve, or pathology in the forearm mimicking CTS when the electrodiagnostic (EDX) studies are nonlocalizing [4,5]. Measuring the cross-sectional area (CSA) of the median nerve at the CT (carpal tunnel) inlet has become the standard sonographic parameter for documenting entrapment at the CT [6]. Most often, the trace technique is used to delineate the circumference of the median nerve within the hyperechoic epineurium. The cut-off value lacks uniformity due to a paucity of standardized normative reference values [7].

In Roll et al.’s meta-analysis of data from 73 studies from healthy individuals for US reference values for median nerve CSA, the median nerve CSA was 6.46 mm^2^, 8.68 mm^2^, and 8.6 mm^2^ at the proximal forearm, the CT inlet, and the proximal CT, respectively [8]. The ratio of the median nerve CSA at the wrist compared to the forearm (wrist/forearm ratio) is another criterion, with a cut-off value of 1.4 [9,10]. With median nerve enlargement at the wrist, as seen in CTS, a larger ratio is anticipated. In most cases of moderate to severe CTS, the median nerve CSA ranges from 14 to 25 mm^2^ but rarely exceeds 30 mm^2^. Terms such as “mega” and “giant” median nerves have been used in the literature to denote median nerves with a CSA of 19–30 mm^2^ [1,11]. Only a few reports have highlighted this phenomenon of mega and giant median nerves [1,11,12,13,14]. The underlying mechanism of such marked enlargement in some patients with CTS is speculative, and factors such as a patient’s age, body mass index (BMI), and duration of median nerve entrapment are considered. CTS may not predispose to a significant increase in CSA in certain conditions, such as advanced age [15] and hereditary transthyretin amyloidosis (ATTRv) [16,17].

In this report, we evaluate the clinical, EDX, and US features of patients with a median nerve CSA of 40 mm^2^ or more (denoted as markedly enlarged median nerve) at the wrist. We also discuss the possible pathophysiologic mechanisms associated with markedly enlarged median nerve at the wrist.

## 2. Materials and Methods

Our AANEM-accredited Neurodiagnostic Center evaluates roughly 1000 patients each year who are referred for EDX studies mostly by hand surgeons and neurosurgeons. Under an Institutional Review Board (IRB)-approved protocol, we performed an 11-year (4 February 2014–24 April 2025) retrospective analysis of US data collected from patients referred for EDX studies to confirm the diagnosis of CTS. Of 4584 patients with CTS who underwent an US along with EDX studies, there were 36 (0.78%) patients in whom the CSA of the median nerve at the wrist was 40 mm^2^ or greater. The EDX protocol included nerve conduction and needle EMG studies. The US protocol included studying long-axis and short-axis images of the median nerve at the wrist and the forearm using an 8–18 or 6–15 Mhz probe. All CSA measurements were made by a single observer.

### 2.1. Inclusion and Exclusion Criteria

Inclusion criteria were patients with a median nerve CSA at the wrist of 40 mm^2^ or greater. We selected 40 mm^2^ as an arbitrary cut-off for a markedly enlarged median nerve. This number was chosen since most cases in the literature reported a number below 40 mm^2^ for the median nerve CSA. In the current literature, there is only minimal discussion regarding a median nerve with a CSA ≥ 40 mm^2^. Exclusion criteria included patients with diffuse enlargement of the median nerve (at the wrist as well as the forearm). Several metrics were collected, including the patients’ gender and age, laterality (left/right/bilateral), duration of symptoms, diabetes mellitus, BMI, clinical findings (sensory loss and thenar atrophy), and severity of the median nerve entrapment at the CT by EDX findings. BMI values were recorded based on height and weight on the date of the EMG and US studies.

Median nerve motor conduction velocity across the elbow, forearm, and wrist was determined using the standard protocol in our lab [18]. The EDX study was performed by placing the recording electrode over the abductor pollicis brevis (APB) muscle or the 2nd lumbrical muscle if no compound muscle action potentials (CMAPs) were noted over the APB muscle. Sensory conduction was studied with the recording electrode over the index finger and antidromic stimulation at the wrist. US studies were conducted on all patients, with documentation of the CSA at the wrist and forearm.

An analysis comparing two groups of patients with CTS was also performed: (1) those with a median nerve CSA between 12 and 20 mm^2^ and (2) those with a median nerve CSA of 40 mm^2^ or greater. Several characteristics were evaluated for this comparison, including patients’ age and gender, laterality (left/right/bilateral), duration of symptoms, diabetes mellitus, BMI, clinical findings (thenar atrophy), and severity of the median nerve entrapment at the CT by EDX findings.

### 2.2. Statistical Analysis

Patients with a median nerve CSA at the wrist between 12 and 20 mm^2^ were compared to our study population of patients with a wrist CSA equal to or greater than 40 mm^2^ using ANOVA, Kruskal–Wallis, and Chi-squared or Fisher’s exact tests as appropriate, with a *p*-value ≤ 0.05 defining statistical significance. Demographics and disease traits were summarized only for the patients with a wrist CSA 40 mm^2^ or greater. These tests were conducted using R version 4.2.3 (15 March 2023 ucrt).

### 2.3. Institutional Review Board Approval of Research

Informed consent was obtained from all patients. The WCG IRB determined that our study was exempt under 45 CFR 46.104(d)(4). The IRB number is 20250164, and the Ethic Approval Code is 01172025. The IRB approval date was 17 January 2025.

## 3. Results

### 3.1. Demographics

A total of 36 patients had evidence of a markedly enlarged median nerve detected by US (Table 1). The mean age was 52.94 years (SD = 13.83 years), and the majority (24 [66.7%]) of patients were female. The markedly enlarged median nerve was more common on the right side (23 [63.9%]), followed by the left side (10 [27.8%]) and bilaterality (3 [8.3%]). Thirty-four (94.4%) patients were right-hand dominant. The markedly enlarged median nerve side corresponded to the dominant hand in 27 (75.0%) patients. Seven (19.4%) patients had diabetes mellitus. The mean BMI was 36.48 (SD = 8.49). The median duration of symptoms of CTS was 13.50 years (IQR = 5.00, 20.00).

### 3.2. Clinical Features

**Figure 1:**
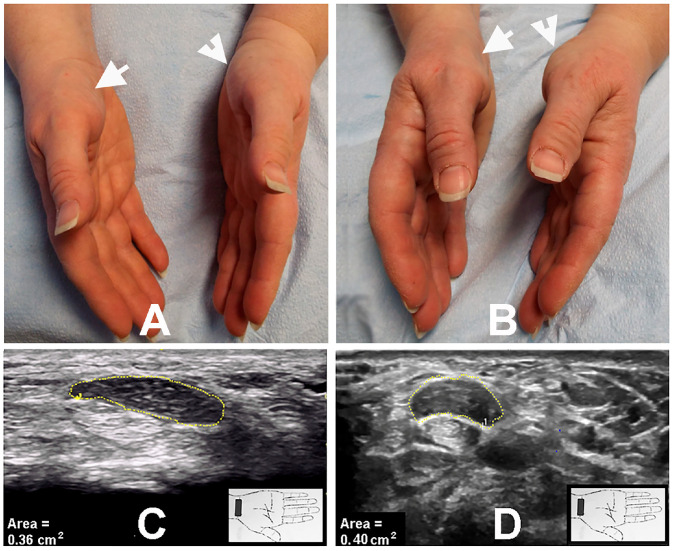
(**A**) Mild thenar atrophy (arrows). (**B**) Marked thenar atrophy 6 years later (arrows). (**C**) CSA at carpal tunnel inlet of 35 mm^2^. (**D**) CSA increased to 40 mm^2^ 6 years later. CSA: cross-sectional area.

**Figure 2:**
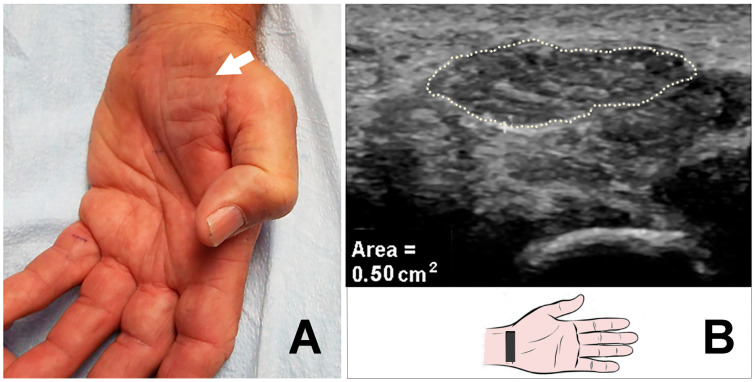
(**A**) Severe thenar muscle atrophy. (**B**) Massively increased CSA (50 mm^2^) of the median nerve (arrow) at carpal tunnel inlet. CSA: cross-sectional area.

The markedly enlarged median nerve was detected by US in a total of 39 hands (36 patients, with 3 bilateral) (Table 2). Of the 39 hands, thenar atrophy was observed in 15 (38.5%) hands, and pinprick loss was noted in all 39 (100%) hands (Figure 1A–D, Figure 2A,B). Of the 39 hands, 3 (7.7%) hands had previously undergone a carpal tunnel release (CTR) procedure.

One nondiabetic nonsmoking patient had evidence of painless nonhealing ulceration of the left middle finger, with a history of ulceration in the left index and middle fingers resulting in amputation of the tip of the index finger [19]. This patient was diagnosed with “ulcerative/mutilating CTS”. Another patient had a history of acromegaly.

### 3.3. Electrodiagnostic Studies

The severity of median nerve entrapment at the CT based on the EDX studies is depicted in Table 3. Moderately severe and severe median nerve entrapment at the CT was noted in 21 (53.8%) and 16 (41.0%) hands, respectively. Only two (5.1%) hands had moderate entrapment, and none had mild entrapment.

A total of 12 (30.8%) hands had no CMAPs over the APB and the second lumbrical muscles (Table 4). The distal motor latency of the APB muscle was greater than 6.0 ms in 28 (75.7%) hands. The motor unit recruitment was decreased in 29 (74.4%) hands and absent in 7 (17.9%) hands. Fibrillations and positive waves in the APB muscle were detected in 12 (30.8%) hands. Sensory nerve action potentials (SNAPs) were not detected in 31 (79.5%) hands (Table 5).

### 3.4. Ultrasound Studies

**Figure 3:**
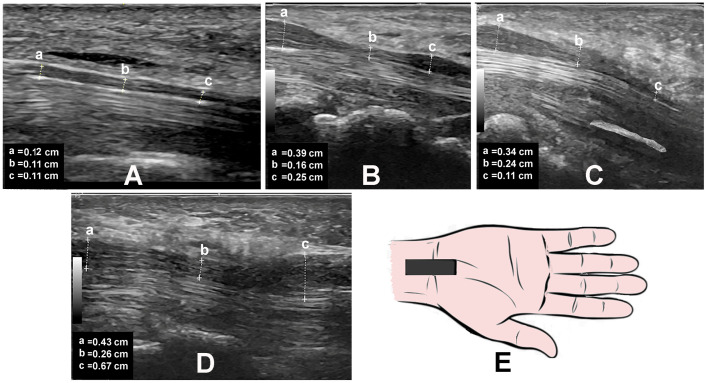
Normal median nerve: (**A**) Long-axis view at the wrist of an asymptomatic person showing normal median nerve at the carpal tunnel inlet, within the carpal tunnel, and at the carpal tunnel outlet with almost the same diameter (“rail track”). Morphological patterns of the markedly enlarged median nerve at the wrist. (**B**) Equal increase in diameter at the carpal tunnel inlet and outlet (“hourglass”). (**C**) Maximum diameter at carpal tunnel inlet (“rat tail”). (**D**) Maximum diameter at carpal tunnel outlet (“snake head”). (**E**) Hand-drawn depiction of the location of the ultrasound probe.

The mean wrist CSA was 45.41 mm^2^ (SD = 5.59) (Figure 3A–C) (Table 6). The wrist CSA was between 40 and 44 mm^2^ in 20 (51.3%) hands, between 45 and 49 mm^2^ in 13 (33.3%) hands, and 50 mm^2^ or greater in 6 (15.4%) hands. The mean forearm CSA was 9.08 mm^2^ (SD = 2.23), with 34 (82.9%) hands having a forearm CSA greater than 10 mm^2^. The mean wrist/forearm ratio was 5.20 (SD = 1.325); 22 (56.4%) hands had a mean wrist/forearm ratio of 5 or greater. The markedly enlarged median nerve often tapers and shows a normal or mild increase in CSA at the mid-forearm (Table 6, Video S1, and Figure S1).

### 3.5. Comparison of Patients with Carpal Tunnel Syndrome Accompanied by a Median Nerve Cross-Sectional Area Between 12 and 20 mm^2^ or 40 mm^2^ and Greater

**Figure 4:**
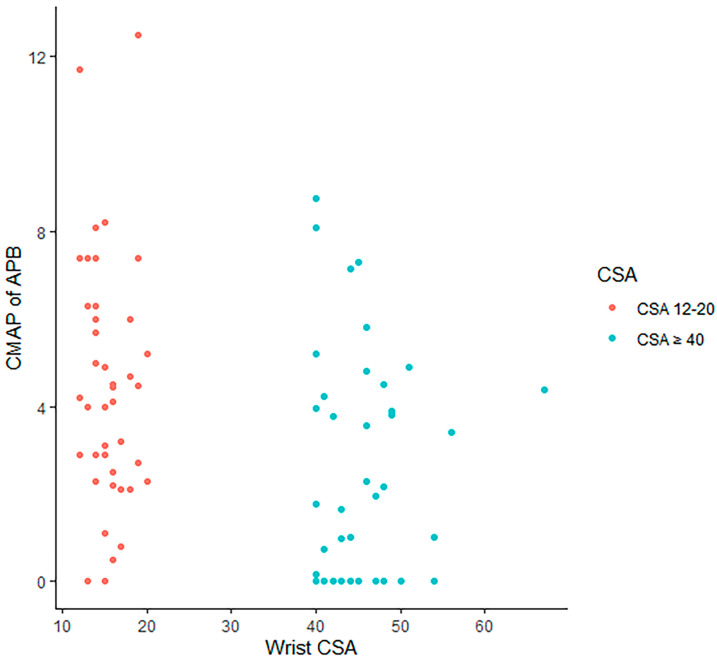
A scatterplot demonstrating the distribution of the wrist CSA by CMAPs, comparing patients with a wrist CSA 12–20 mm^2^ and those with a wrist CSA ≥ 40 mm^2^.

**Figure 5:**
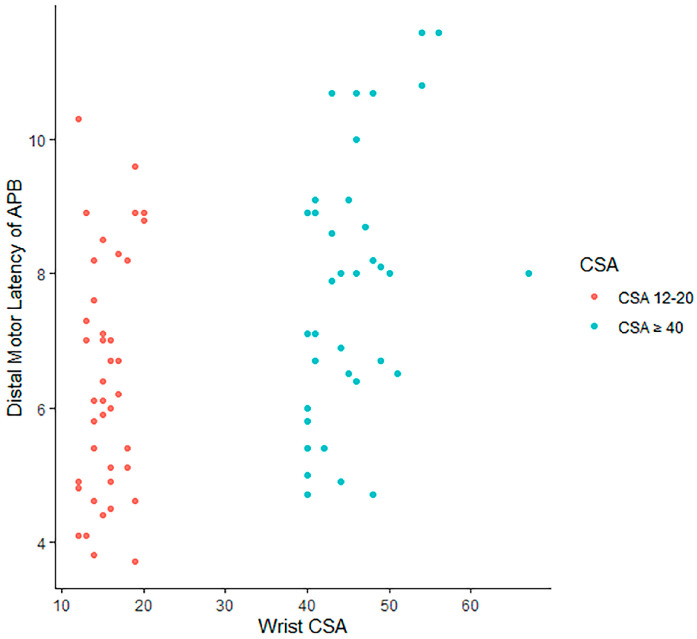
A scatterplot revealing the distribution of the wrist CSA by motor latency, comparing patients with a wrist CSA 12–20 mm^2^ and those with a wrist CSA ≥ 40 mm^2^.

The mean age, median duration of symptoms, mean BMI, presence of thenar atrophy, and severity of CTS by EDX studies were significantly different between patients with a median nerve CSA between 12 and 20 mm^2^ and those with a median nerve CSA of 40 mm^2^ and greater (Table 7). The patients with a CSA 40 mm^2^ and greater were younger (52.94 vs. 63.39 years [*p* = 0.002]) and had a longer symptom duration (13.50 vs. 1.00 years [*p* < 0.001]), higher BMI (36.48 vs. 28.61 [*p* < 0.001]), thenar atrophy (15 [38.5%] patients vs. 2 [4.9%] patients [*p* = 0.001]), and more severe CTS, according to EDX studies (16 [41.0%] patients with severe CTS vs. 3 [7.3%] patients with severe CTS [*p* < 0.001]). Gender, side of symptoms, and diabetes mellitus were not statistically significant between the two groups. The scatterplots demonstrate the distribution of wrist CSA by CMAPs (Figure 4) and motor latency (Figure 5), comparing patients with a wrist CSA 12–20 mm^2^ and those with a wrist CSA ≥ 40 mm^2^.

## 4. Discussion

A few case reports have highlighted the phenomenon of mega and giant median nerves [1,11,12,13,14]. Tram and Vitale described two cases of giant median nerves by US, with the first case having a right median nerve CSA of 36.2 mm^2^, while the second patient had bilateral giant median nerves (left: CSA 31.3 mm^2^; right: 32.4 mm^2^) [1]. These authors encouraged the use of US as a first-line modality in the diagnostic evaluation of CTS [1]. Chabok reported a case with a median nerve width of 25 mm that was noted during the CTR, as the patient had only undergone an MRI and not an US preoperatively [14]. The patient subsequently underwent a second CTR on the contralateral side months later, which also revealed a similar-sized median nerve.

In Kohls and colleagues’ study of patients who presented with upper extremity paresthesia, two groups were established: (1) “mega” median nerves with a CSA of the median nerve > 2 SD above average [CSA ≥ 19 mm^2^]) (25 median nerves) and (2) “nonmega” median nerves [CSA < 19 mm^2^] (400 median nerves) [11]. Mega median nerves were associated with a higher patient weight (223 vs. 196 pounds) and BMI (36.9 vs. 31.9), diabetes mellitus, more altered EDX findings (primarily motor latency and amplitude), and a worse Boston Carpal Tunnel Questionnaire Symptom Severity Scale score compared to nonmega median nerves. The main difference between Kohls et al.’s study and ours is the substantial difference in the mega/massive median nerve CSA (CSA ≥ 19 mm^2^ in theirs vs. ≥ 40 mm^2^ in ours).

US imaging of the median nerve is valuable in the diagnosis and management of CTS. When both CMAPs and SNAPs are absent, US is crucial in confirming the diagnosis and ruling out a more proximal lesion of the median nerve. US enables detection of marked enlargement, as well as abnormalities such as bifid median nerve, persistent median artery, and other causes of compression of the nerve which may provide diagnostic and prognostic clues. In Ahisha and Paker’s study of US-guided steroid injections in patients with CTS and bifid median nerves, the response to corticosteroid injections was significantly lower in patients with bifid median nerves compared to normal median nerves in CTS [20]. These authors suggested using alternative injection options or combination therapies [20]. Only one patient had a bifid median nerve in the current series. US is also useful in visualizing the anatomical variations of the recurrent motor branch of the median nerve, which is important during a CTR [21]. There is currently no sufficient data to formulate treatment options, including the ideal surgical approach, for patients with CTS and a markedly enlarged median nerve.

Several mechanisms may underlie the development of a markedly enlarged median nerve. In our study, the US showed edema, fibrous tissue proliferation, and loss of fascicles in the markedly enlarged median nerves. In advanced CTS, an hourglass appearance due to constriction of the median nerve is a well-known observation, specifically including swelling proximal and distal to the site of compression, with proximal swelling being more common [22]. The long-term pressure within the CT may result in cytokine-induced cell damage, interleukin (IL)-6 mediated fibroblast proliferation, and fibrosis [1,23,24]. As the mean duration of CTS symptoms in our study was 15 years, this represented an extended duration of compression of the median nerve at the CT, which could account for the substantially larger size of the median nerve. It is possible that the markedly enlarged median nerve may itself have contributed to more severe entrapment at the CT. The median nerves were normal or mildly enlarged at the forearm compared to the wrist, as evidenced by the high wrist/forearm ratio in most cases. It appeared that the marked enlargement at the CT inlet tapered as the nerve was traced proximally (Video S1). Conditions like hereditary neuropathy with liability to pressure palsies (HNPP) caused by mutations in the PMP22 gene (usually a deletion of one copy) [25,26], may cause diffuse enlargement of the median nerve and were a consideration in 3 patients in our study with bilateral markedly enlarged median nerves. Since these patients did not have any history of prior episodes of involvement of other nerves such as the peroneal (causing foot drop) or radial (causing wrist drop), we did not consider genetic testing. None of our patients were also evaluated for ATTRv.

Motor nerve conduction velocity (NCV) provides insight into the status of myelination but not necessarily the degree of axon loss (except by the size of CMAPs). The extent of axon loss can only be determined by needle EMG (presence of fibrillations and altered motor unit morphology and recruitment pattern). This is crucial information with a prognostic implication and is not provided by simply performing NCV or US evaluation. In extreme enlargement of the median nerve, needle EMG provides data on the extent of axon loss. This information may provide further insight into the underlying pathophysiology with prospective studies.

### Strengths and Limitations

The strength of the present study is that it features a large number of patients with a markedly enlarged median nerve detected by US studies, focusing on the clinical and EDX features of this uncommon phenomenon. By identifying a markedly enlarged median nerve during the evaluation of CTS, appropriate and timely management can be pursued, and particular attention should be paid to this rare phenomenon intraoperatively to avoid potential nerve injury. The limitations of this study include its retrospective nature and the lack of follow-up to assess how the markedly enlarged CSA of the median nerve at the wrist changes the outcome after CTR. Another limitation is the lack of data on the mobility of the median nerve during wrist movements and the vascularity by Doppler studies.

## 5. Conclusions

Although rare, surgeons should be aware of the occurrence of markedly enlarged median nerves at the CT, which may be important during a CTR. US evaluation of the median nerve at the CT should be an integral part of the work-up of patients with thenar muscle atrophy and loss of CMAPs and SNAPs on EDX studies.

## Figures and Tables

**Table 1:** Clinical Findings of patients with a markedly enlarged median nerve by ultrasound.

Characteristics	Number of Patients (*n* = 36)
Age (mean (SD))	52.94 (13.83)
Sex (%)	
Male	12 (33.3)
Female	24 (66.7)
Side (%)	
Right	23 (63.9)
Left	10 (27.8)
Both	3 (8.3)
Hand Dominant (%)	
Right	34 (94.4)
Left	2 (5.6)
Hand dominance corresponds with side of symptoms (%)	
No	9 (25.0)
Yes	27 (75.0)
Diabetes mellitus (%)	
No	29 (80.6)
Yes	7 (19.4)
Body Mass Index (mean (SD))	36.48 (8.49)
Body Mass Index Categorical (%)	
<25	2 (5.6)
25–29.9	7 (19.4)
30–39.9	14 (38.9)
>40	13 (36.1)
Duration (median [IQR]) (years)	13.50 [5.00, 20.00]
Duration Categorical (%) (years)	
<1	2 (5.6)
1–5	8 (22.2)
>5	22 (61.1)
Unknown	4 (11.1)

**Table 2:** Neurological examination of patients with a markedly enlarged median nerve by ultrasound (total number of hands).

	Number of Hands (*n* = 39)
Thenar atrophy = Yes (%)	15 (38.5)
Sensation decreased/absent = Yes (%)	39 (100.0)
History of carpal tunnel release = Yes (%)	3 (7.7)

**Table 3:** Severity of median nerve entrapment at the carpal tunnel by electrodiagnostic findings of patients with a markedly enlarged median nerve by ultrasound (total number hands).

Grade CTS (%)	Criteria	Number of Hands (*n* = 39)
Mild	Only sensory fascicles affected	0 (0.0)
Moderate	Sensory and motor fascicles affected	2 (5.1)
Moderately Severe	Sensory and motor fascicles affected with motor unit changes in the APB	21 (53.8)
Severe	Loss of SNAPs, loss/very small amplitude of CMAPs, loss of CMAPs and SNAPs, denervation of APB	16 (41.0)

CTS: carpal tunnel syndrome. APB: abductor pollicis brevis muscle. SNAPs: sensory nerve action potentials. CMAPs: compound muscle action potentials.

**Table 4:** Details of motor electrodiagnostic findings in carpal tunnel syndrome in patients with a markedly enlarged median nerve by ultrasound (total number of hands).

	Number of Hands (*n* = 39)
CMAP (mean (SD))	3.53 (2.13)
CMAP Categorical (%)	
<1 mV	3 (7.7)
1–1.99 mV	5 (12.8)
2–4 mV	8 (20.5)
>4 mV	11 (28.2)
Absent	12 (30.8)
Distal latency (mean (SD))	7.75 (2.01)
Distal motor latency of APB (%)	
≤6 ms	9 (24.3)
>6 ms	28 (75.7)
Motor unit recruitment (%)	
Normal	3 (7.7)
Decreased	29 (74.4)
Absent	7 (17.9)
Fibrillations/Positive waves (%)	
No	27 (69.2)
Yes	12 (30.8)

CMAPs: compound muscle action potentials. APB: abductor pollicis brevis. Normal cut-off values: CMAP ≥ 5 mV.

**Table 5:** Details of sensory electrodiagnostic findings in carpal tunnel syndrome in patients with a markedly enlarged median nerve by ultrasound (total number of hands).

	Number of Hands (*n* = 39)
SNAP amplitude (median [IQR])	5.80 [5.40, 5.80]
SNAP amplitude Categorical (%)	
>25 μV	2 (5.1)
10–25 μV	1 (2.6)
<10 μV	5 (12.8)
Absent	31 (79.5)
SNAP latency (median [IQR])	6.80 [5.10, 7.50]
SNAP latency (%)	
Absent	31 (79.5)
>6.0 ms	1 (2.6)
3.5–6.0 ms	7 (17.9)

SNAPs: sensory nerve action potentials. Normal cut-off values: SNAP ≥ 25 μV.

**Table 6:** Ultrasound findings of the median nerve in patients with a markedly enlarged median nerve by ultrasound (total number of hands).

	Number of Hands (*n* = 39)
Wrist CSA (mean (SD))	45.41 (5.59)
Wrist CSA (%)	
40–44 mm^2^	20 (51.3)
45–49 mm^2^	13 (33.3)
≥50 mm^2^	6 (15.4)
Forearm CSA (mean (SD))	9.08 (2.23)
Forearm CSA (%)	
≤10 mm^2^	32 (82.1)
>10 mm^2^	7 (17.9)
Wrist/Forearm Ratio (mean (SD))	5.20 (1.25)
Wrist/Forearm Ratio (%)	
<5	17 (43.6)
≥5	22 (56.4)

CSA: cross-sectional area.

**Table 7:** Comparison between patients with carpal tunnel syndrome with a median nerve cross-sectional area between 12 and 20 mm^2^ and those with a median nerve cross-sectional area of 40 mm^2^ and greater.

	CSA 12–20 mm^2^	CSA ≥ 40 mm^2^	*p*-Value
n Subjects	38	36	
n Hands	41	39	
Age (mean (SD))	63.39 (14.69)	52.94 (13.83)	0.002 ^1^
Sex (%)			0.876 ^2^
Male	11 (28.9)	12 (33.3)	
Female	27 (71.1)	24 (66.7)	
Side (%)			0.875 ^3^
Right	22 (57.9)	23 (63.9)	
Left	13 (34.2)	10 (27.8)	
Both	3 (7.9)	3 (8.3)	
Duration (median [IQR])	1.00 [0.42, 5.00]	13.50 [5.00, 20.00]	<0.001 ^4^
Duration (%)			<0.001 ^2^
<1 year	10 (24.4)	2 (5.1)	
1–5 years	8 (19.5)	8 (20.5)	
>5 years	3 (7.3)	22 (56.4)	
Unknown	20 (48.8)	7 (17.9)	
Diabetes mellitus (%)			0.185 ^3^
No	35 (92.1)	29 (80.6)	
Yes	3 (7.9)	7 (19.4)	
BMI (mean (SD))	28.61 (5.43)	36.48 (8.49)	<0.001 ^4^
BMI (%)			0.001 ^3^
<25	7 (18.4)	2 (5.6)	
25–29.9	19 (50.0)	7 (19.4)	
30–39.9	9 (23.7)	14 (38.9)	
>40	3 (7.9)	13 (36.1)	
Thenar atrophy (%)			0.001 ^2^
No	39 (95.1)	24 (61.5)	
Yes	2 (4.9)	15 (38.5)	
Grade CTS (%)			<0.001 ^3^
Mild	4 (9.8)	0 (0.0)	
Moderate	8 (19.5)	2 (5.1)	
Moderately Severe	26 (63.4)	21 (53.8)	
Severe	3 (7.3)	16 (41.0)	

^1^ = ANOVA; ^2^ = Chi-squared test; ^3^ = Fisher’s Exact Test; and ^4^ = Kruskal–Wallis Test. CSA: cross-sectional area. BMI: body mass index. CTS: carpal tunnel syndrome.

## Data Availability

All of the data for this study is included in the current article.

## Supplementary Materials

The following supporting information can be downloaded at: https://www.mdpi.com/article/10.3390/neurolint17080124/s1, Figure S1: Ultrasound position of the probe for Video S1; Video S1: Ultrasonography of the median nerve from the mid forearm to the carpal tunnel inlet.
