# Supplementary material for: Clinical and Electrodiagnostic Correlations of Ultrasound-Detected Markedly Enlarged Median Nerve at the Wrist
Source: Neurol Int. 2025 Aug 7;17(8):124. doi: 10.3390/neurolint17080124 (PMC12389687; doi:10.3390/neurolint17080124)
Supplement: Supplementary file 1 [file neurolint-17-00124-s001.zip › neurolint-3741793-supplementary.pdf]

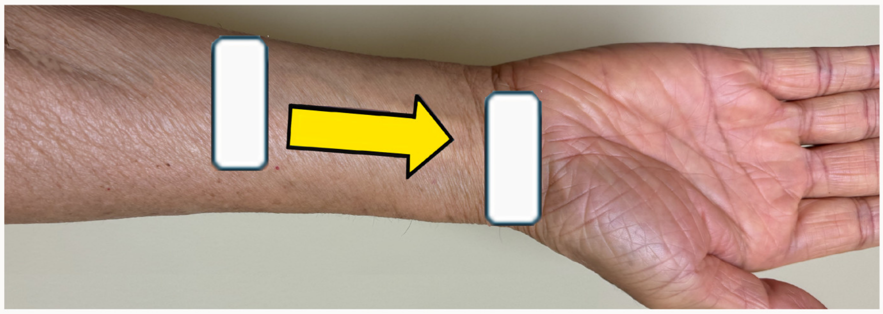

Figure S1. Ultrasound position of the probe for Video S1. The rectangles represent the positions of the ultrasound probe. The arrow shows the direction of moving the probe to obtain the video from the mid forearm to the wrist.

Video S1. Ultrasonography of the median nerve from the mid-forearm to the carpal tunnel inlet showing a normal cross-sectional area (CSA) at the forearm, a gradual increase in CSA at the distal forearm, and maximum CSA at the wrist. The median nerve appears hypoechoic and markedly enlarged at the CT inlet (red arrow).
